# The Governance of Indigenous Natural Products in Namibia: A Policy Network Analysis

**DOI:** 10.1007/s00267-017-0968-3

**Published:** 2018-01-09

**Authors:** Albertina Ndeinoma, K. Freerk Wiersum, Bas Arts

**Affiliations:** 1 Department of Integrated Environmental Science, University of Namibia, P/Bag 5520, Oshakati, Namibia; 2 Forest and Nature Conservation Policy Group, Wageningen University and Research Centre, 6700 AA Wageningen, The Netherlands

**Keywords:** Indigenous natural products, Policy network, Non-timber forest product, Actor relations, Governance structure, Sustainable commercialization

## Abstract

At the end of the 20th century, optimism existed that non-timber forest products (NTFPs) can form an integral part in conservation and development strategies. However, there is limited knowledge on how the different stakeholders could relate to the state or to each other in promoting commercialization of NTFPs. Applying the policy network as an analytical framework, we investigated the structural patterns of actor relations in the governance structure of indigenous natural products (INPs) in Namibia, to understand the implications of such relations on INP policy process. The findings indicate that the INP policy network in Namibia is multi-dimensional, consisting of the Indigenous Plant Task Team (IPTT)—the key governance structure for resource mobilization and information sharing; and functional relations which serve specific roles in the INP value chain. The existing relations have facilitated policy development particularly for heavily regulated species, such as devil’s claw; but for other species, only incremental changes are observed in terms of small-scale processing facilities for value addition and exclusive purchase agreements for sustainable sourcing of INPs. Participation of primary producers, private actors and quality standardization bodies is limited in INPs governance structures, which narrow the scope of information sharing. Consequently, despite that the IPTT has fostered publicly funded explorative pilot projects, ranging from production to marketing of INPs, there are no clear guidelines how these projects results can be transferred to private entities for possible commercialization. Further collaboration and information sharing is needed to guide public sector relations with the private entities and cooperatives.

## Introduction

At the end of the 20th century optimism existed that non-timber forest products (NTFPs) could contribute toward combined goals of conservation and development (Belcher et al. 2005; Kusters et al. 2006). These studies concluded that in order to achieve these combined goals, a focus on multi-stakeholder governance is required to foster a multi-disciplinary engagement and inter-sectoral policies integration (Arnold and Ruiz Pérez (2001). The governance of NTFPs involves diverse issues including resource management and marketing systems, which are often directed at specific species. This is reflected in a great diversity in institutions for both access to NTFP resources and markets (Laird et al. 2010; Ros-Tonen and Kusters 2011; Wiersum et al. 2014).

The complexity of NTFP governance is well reflected in Namibia. The promotion of NTFPs—in Namibia locally known as indigenous natural products (INPs)—involves actors from the state, NGOs, private sectors, and local communities. Initially, isolated stakeholders conducted commercialization trials of promising indigenous species, such as devil’s claw (*Harpagopytum* spp.), marula (*Sclerocarya birrea*), Kalahari melon (*Citrullus lanatus*), and silk from the African moth (*Gonometa postica*)—also known as Kalahari wild silk (Ministry of Agriculture Water and Rural development 2003). Through these pilot projects, specific governance arrangements were developed for the different species. For instance, in 1999 a decision was made to establish a Devil’s Claw Working Group (DCWG) as a forum for developing policies to regulate harvesting and trade of devil’s claw. The DCWG also served as focal point for international consultations, specifically the proposals to list devil’s claw under the Convention on International Trade of Wild Fauna and Flora (CITES).

In order to further coordinate the different types of INPs, Namibia also established in 2000 the Indigenous Plant Task Team (IPTT)—a multi-stakeholder forum with representatives from the government and non-government entities for coordinating the implementation of the action strategy for all INPs (du Plessis 2007). The implementation of the IPTT action strategies and plans took a new governance approach, which encourages stakeholder’s participation in pilot scale projects for sustainable commercialization of potential products. In fulfilling its commercialization roles, the IPTT also engages in the provision of access to genetic resources from indigenous plants, thereby contributing to the national debate on access and benefit sharing, which is provided under the Convention on Biological Diversity (CBD). In 2007 an Interim-bio Prospecting Committee (IBPC) was established to strengthen attention to issues of access and benefit sharing in the use of genetic resources.

Gradually a complex governance structure has emerged composed of several multi-stakeholder governance bodies for policymaking, implementation, and information exchange. Namibia has been commented for establishing the IPTT, a multi-stakeholder body through which INP activities are coordinated (Laird et al. 2010).

However, the relationship between the stakeholders in these different governance bodies has not been systematically assessed to understand interactive relations between the different actors and the influence of these relations on INP policy outcomes. Specifically, the IPTT has over the years failed to establish an enterprise ownership model, through which business opportunities generated with public funds can be transferred to the private entities. Also, the roles and functions of the IPTT has been too broad, including both pilot processing; technology development; product research and development as well as marketing and promotion. Differentiation of activities can be guided by a clear understanding of relational patterns in governance structures.

This paper analyses the structure of the policy network and the interactions between stakeholders in the Namibian INP policy sector by (i) identifying the different *governance bodies* that emerged in Namibia to coordinate the governance of INPs, (ii) assessing the structural relations among the different actors in the INP sector, and (iii) analyzing the implications of these structural relations of INP governance on the policy process. This analysis will provide an understanding on the structural arrangements through which collective actions for INP management and policies have emerged and the roles of different actor groups in the different governance bodies. Furthermore, it will consider how interactions between different groups of INP actors and different components of the governance structure influence the policy outcomes. The following research questions will be addressed:

1. Which formal bodies for INP governance have been established in Namibia and what are their structural relations in terms of stakeholder representation?
2. What functional relations exist between the stakeholders represented in the various governance bodies?
3. How have the lessons learned from the various activities in the multi-dimensional governance structure influenced INP policy development?

Following this introductory section we give a brief background of the policy network approach which is used in the paper to analyze structural and functional patterns of the INP governance network. We then present the method that was followed to collect and analyze the data for this paper. The research questions are addressed in the consecutive sections, after which the last section discusses the implications of the INP governance structure on INP policy development and implementation in Namibia.

## Theoretical Framework

The institutional structures for environmental governance have recently changed from government to governance (Lemos and Agrawal 2006; Rhodes 1997; Young 2009). These changes are a result of increasing societal differentiation in terms of specialization in functional roles and dispersion of resources among public, civil, and private actors (Schneider 1992). One major tool to analyze the structure of governance and to understand the role of these structures in the policy process and outcome is the policy network analysis approach (Marin et al. 1991; Rhodes and Marsch 1992). There are two different but closely entwined approaches to policy network analysis in the literature (Börzel 1998).

The structural approach focuses on a policy network as a social structure through which governance is conducted (Marin et al. 1991; Schneider 1992; Torfing 2005). It considers a policy network as a specific arrangement in which multiple actors are represented to participate in the process of decision-making and implementation. The policy network is regarded as a non-hierarchical public–private structure through which resources and technical knowledge is shared between autonomous but inter-dependent actors (Marin et al. 1991; Schneider 1992). This public–private governance network is composed of public actors, private firms, interest organizations (e.g. user group associations, small-scale farmers, trade associations, etc.). In this arrangement, policies do not emerge from concerted and programmed actions at government level, but rather from interactions between these actors (Kenis and Schneider 1999). Thus, in the structural approach, a policy network is conceptualized as a devise to enable resource mobilization, as well as sharing specialized technical information and knowledge relevant for decision-making (Marin et al. 1991; Schneider 1992; Torfing 2005). This perspective does not only focus on the factors leading to joint policymaking, but more importantly considers the structures and processes through which joint policy is organized (Börzel 1998).

The structural approach to policy network analysis has been criticized at several points. In the first place, it has been argued that policy network analysis tends to focus too much on the interactive processes and related institutions, while neglecting the substantive aspect of the specific problem in specific policy domains (Koppenjan and Klijn 2004). Consequently, in addition to analyzing the structural relations in the INP governance network, we also assess the substantive issues involved and the functional relations between the various actors.

Another criticism is that the structural policy network does not explain clearly how interactions between actors in the policy network influence policy process and outcomes (Börzel 1998; Dowding 1995) or to capture the changes that characterize the interactions in the policy process (Dowding 1995; Klijn and Koppenjan 2000). To address these issues, some studies on policy network analysis therefore focus specifically on interest intermediation, examining power relations between the state and the industry, or between the different levels of the state at, for instance, the national and local level (Rhodes 2006; Rhodes and Marsch 1992). This approach gives specific attention to analyzing the distinctive but inter-dependent interests that are represented in the policy network (Börzel 1998). For example, the policy network may be dominated by economic interests, professional interests or government interests. Whereas the economic and professional interests may be focused on specific economic sectors, the government interest ideally serves the interest of the entire society (Marsh and Rhodes 1992).

This paper uses a combination of policy network dimensions to analyze the complexity of the INP governance network in Namibia. It is primarily based on the structural approach, but adds elements of the interest intermediation approach, such as network functions to identify substantive issues and functional relations in the policy network. We also assess how the structural and functional relationships impact on the policy process and outcomes. In making this assessment, we acknowledge that the influence of a policy network on policy outcomes cannot be directly observed and explained as there may be other causal mechanisms which contribute to policy outcomes. Consequently, we use the policy network as an analytical devise to understand the actors’ contributions to the governance process and the pattern of relational linkages between the actors (Parson 2010). This approach assume that there is a dialectic relationship between the policy network as a structure (governance network) and the properties of this structure, i.e. the actors (Toke and Marsh 2003).

**Fig. 1:**
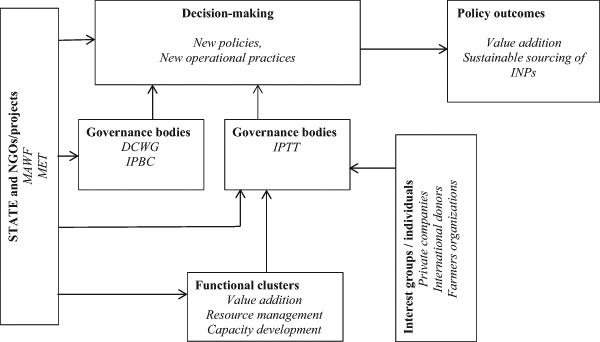
The policy network for the governance of INPs in Namibia

Figure 1 presents the different dimensions of the policy network that exists for INP governance in Namibia. On the one hand, the figure shows a social structure through which decisions are made with participation of interest groups and the state actors. The decisions are either developed into a new policy or new operational activities. On the other hand, the figure also illustrates other dimensions of the INP policy network, such as the type of actors and the functions of different sub-groups of actors.

Through interaction, actors share knowledge and resources and develop a sense of shared understanding and responsibility toward solving complex environmental problems in line with policy objectives. By analyzing a governance network one can understand how relational structures function and how they shape or influence the policy process and policy outcomes.

## Research Methods

**Fig. 2:**
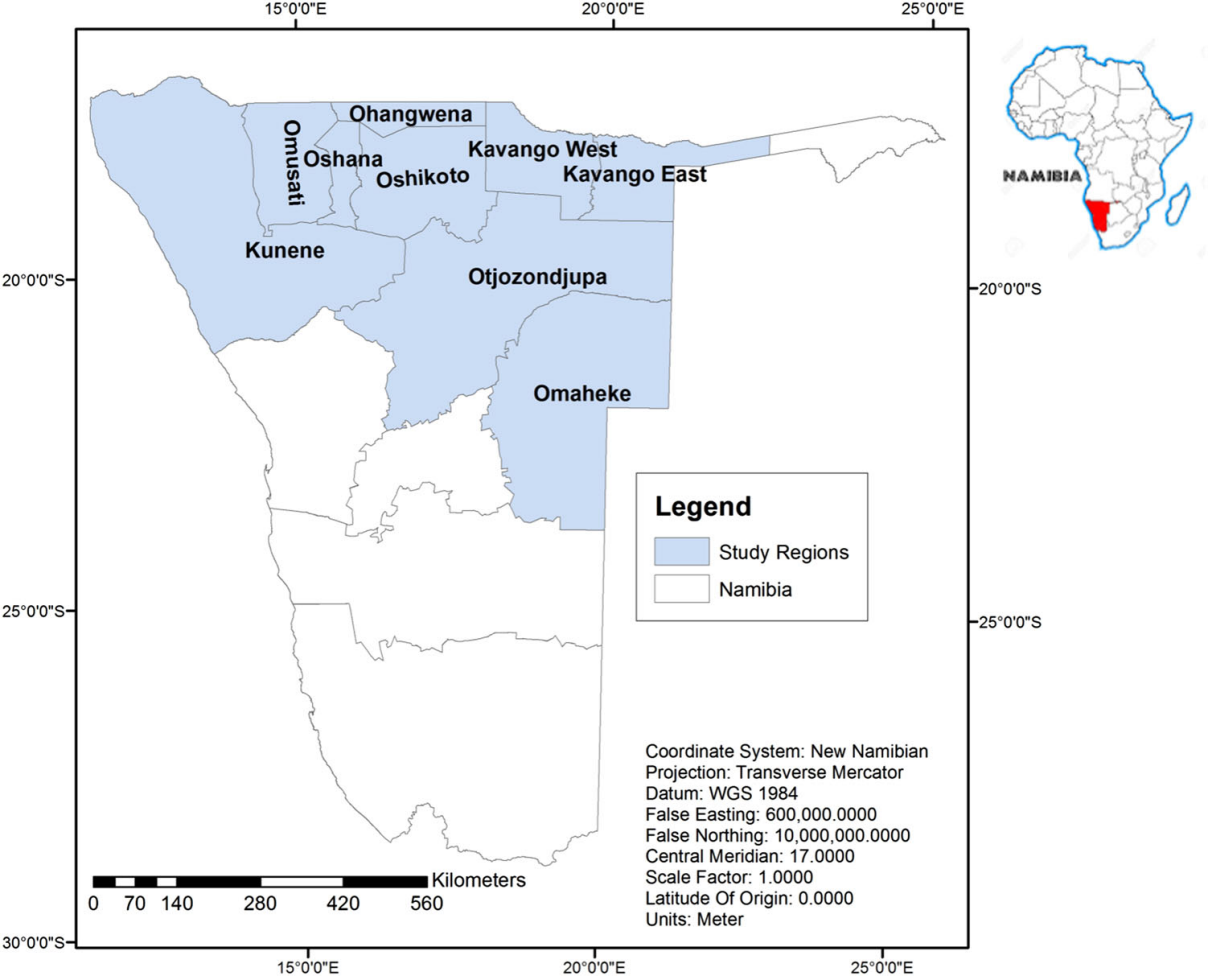
Namibia map showing administrative regions covered for data collection

This study was carried out within the framework of a larger comparative case study on diverse governance arrangements for INPs in Namibia. Data was collected in communal areas of Namibia. Given the spatially distributed nature of INPs in communal areas of Namibia, interviewees were pooled from eight political regions where different types of INPs are harvested, including Omusati, Oshikoto, Ohangwena, Kunene, Oshana, Kavango West and East, Otjozondjupa and Omaheke (Fig. 2). About 55% of the Namibian population live in these regions (National Planning Commission 2012). Communal areas are predominantly rural with high dependence on subsistence farming and natural resources. Communal land is formally state-owned and the occupants use and manage resources in these areas either “privately” (especially within cultivated farm lands) or communally in range lands, communal conservancies and community forests (Mendelsohn et al. 2012). Depending on whether INPs are domesticated on cultivated farm lands, or collected in different types of communal areas, local people have different levels of control (rights) toward the different types of resources (Ndeinoma and Wiersum 2016).

**Fig. 3:**
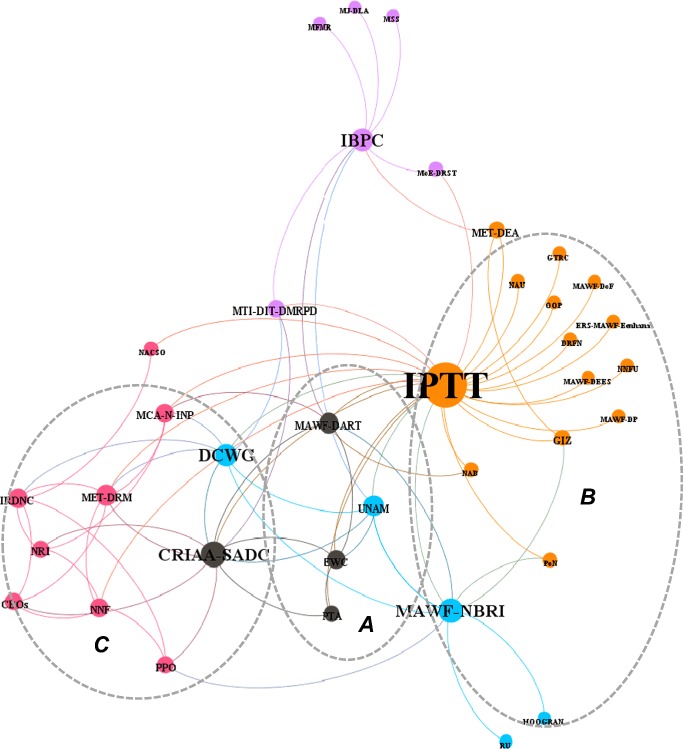
Actor interactions on major functions for implementing INP development activities. (**a**) Product quality, research, standardization, and value addition. (**b**) Resource management assessment and monitoring. (**c**) Institutional capacity development. MET Ministry of Environment and Tourism, DRM Directorate of Resource Management, MAWF Ministry of Agriculture, Water and Forestry, CBO Community based Organizations, CRIAA-SADC Centre for Research Information Action in Africa—Southern African Development and Consulting, DART Directorate of Research, DCWG Devil’s Claw Working Group, DEA Directorate of Environmental Affairs, DEES Directorate of Engineering and Extension Services, DoF Directorate of Forestry, DP Directorate of Planning, DRFN Desert Research Foundation of Namibia, DRM Directorate of Resource Management, ERSC Eco-Regional satellite Centre, EWC Eudafano Women Cooperative, FAO Food Agriculture Organization, GIZ German Development Cooperation, GTRC Gobabeb Training and Research Institute, HOOGRAN Hoodia Growers Association of Namibia, IBPC Interim Bioprospecting Committee, ICEMA/FFEM Integrated Community-based Ecosystem Management/French Funds for Global Environment, IRDNC Integrated Rural Development and Nature Conservation, MAWF Ministry of Agriculture Water and Forestry, MCA-N-INP Millennium Challenge Account—Namibia –Indigenous Natural Product, MET Ministry of Environment and Tourism, MFMR Ministry of Fishery and Marine Resources, MJ-DLA Ministry of Justice—Directorate of Legal Advice, MoE—DRST Ministry of Education-Directorate of Research, Science and Technology, MSS Ministry of Safety and Security, MTI Ministry of Trade and Industry, NAB Namibia Agronomic Board, NACSO Namibian Association of Community-based Natural Resource Management, NBRI Namibia Botanical Research Institute, NNF Namibia Nature Foundation, Namibia National Farmers Union, NRI Natural Resource Institute, OOP Oontanga Oil Products, PoN Polytechnic of Namibia, PPOs Producer Processor Organization, PTA Phytotrade Africa, RU Rudgers University, TTC Tulongeni Twahangana Cooperative, UNAM University of Namibia

Data on the governance structure for the INPs was collected by means of open-ended interviews and analysis of official proceedings (Silverman 2014). A questionnaire was used as an instrument for data collection during interviews. In order to determine structural relations among INP actors, the questionnaire had a list of INP stakeholders, and respondents were asked to select from the list the actors with whom they collaborated either formally or informally. This information, which indicates ties between actors and organizations, was used to establish a governance structural configuration (Fig. 3). Furthermore, in an attempt to assess the power relations between different INP actors, respondents were asked to identify central actors in performing different categories of functions relevant in the INP value chain.

Key informants in the INP sector were interviewed, including government officials, traditional leaders, traders, entrepreneurs, NGOs, and community-based organizations. A total of 50 key informants in the INP sector were interviewed (Table 1). The participants were purposefully selected on the basis of three key criteria, i.e. involvement with the development of different INP species, membership to one or more INP governance bodies, and employment with either government, civil society, or private sector. The selection also took into account the representation of different ecological regions where different types of INPs are located.

**Table 1:** Categories of stakeholders (respondents)

Category of interviewee	Number of interviewees
Government: MET, MAWF, MIT-SMED (formerly known as MTI), NSI,	18
Civil society: (NGOs): CRIAA-SADC, IRDNC, NACSO and NNF	5
International development agencies: MCA-N-INP, GIZ	5
Research institutes: UNAM, NUST	2
Private sector: Ecoso dynamics, Oontanga oils Producers, TTC, EWC, KC-INP-trust, Gamagu cc, Neema Cosmetics	9
Traditional leaders and community-based organizations (CBOs)^a^	13
Total number of respondents	50

Actor’s acronyms

*CBO* community-based Organizations, *CCC* communal conservancy committee, *CFC* Community Forest Committee, *CRIAA-SADC* Centre for Research Information Action in Africa—Southern African Development and Consulting, *EWC* Eudafano Women Cooperative, *GIZ* German Development Cooperation, *IRDNC* Integrated rural development and nature conservation, *KC-INP* Kunene Conservancy-Indigenous natural product trust, *MAWF* Ministry of Agriculture, Water and Forestry, *MCA-N-INP* Millennium Challenge Account-Namibia-Indigenous Natural Product, *MET* Ministry of Environment and Tourism, *MIT* Ministry of Trade and Industry, *MITS-MED* Ministry of Industrialization, Trade and SME development, *NACSO* Namibian Association of Community-based Natural Resource Management, *NGOs* Non-governmental Organizations, *NNF* Namibia Nature Foundation, *NSI* Namibia Standard Institute, *SHDC* Sustainable Harvesting Devil’s Claw, *TTC* Tulongeni Twahangana Cooperative, *UNAM* University of Namibia

^a^ Members of CBOs often include members of the traditional authority

A visual representation of the governance network relations was established by means of *Gephi*, a social network analysis computer program. This program detects actors with frequent interactions (focusing on similar functions) and clusters them in sub-groups of dense connections. The identified functions for governance of INPs include resource management and assessment; value addition, product quality and standardization; and institutional capacity building. In order to validate these functional relations, an in-depth analysis of official documents, such as workshop proceedings, policy documents, and official proceedings for different governance bodies was carried out to analyze the different collaborations and types of activities identified during interviews.

## Formal Governance Bodies for INPs in Namibia

### Development of a Network of Specialized Governance Bodies

At the end of the 20th century, Namibia recognized the need to develop new strategies for coordinating INP commercialization by different stakeholders. In relation to the various policy issues, a number of special governance bodies for coordinating different INP activities were subsequently formed.

The first specialized governance body that was established was the DCWG. This governance body was established in 1999 in response to issues of over-utilization and unsustainable harvesting methods of devil’s claw. This tuberous plant is one of the major commercial INPs is Namibia and is used as an ingredient in pharmaceutical products, veterinary medicine, and herbal tea. In order to encourage sustainable harvesting and trade of this species, the Ministry of Environment and Tourism (MET) organized a national devil’s claw stakeholder’s workshop in 1999 that was attended by a wide range of stakeholders including harvesters, traders, non-governmental organizations (NGOs), and government officials (Ministry of Environment and Tourism 1999). A range of issues were discussed, mainly drawing lessons from the pilot project on Sustainably Harvested Devil’s Claw (SHDC). As a result of this multi-stakeholder workshop, the DCWG was established, with a mandate to coordinate sustainable utilization, monitor trade and develop policies for devil’s claw. Initially the aim was to have representation from relevant ministries, research institutes, NGOs, harvesters, and exporters. However, official proceedings of this working group show that no representatives of traders or harvesters have been attending meetings of this multi-stakeholder forum.

The DCWG was established at an opportune time to address both national and international issues. Just after the formation of the DCWG in 1999, a proposal to list devil’s claw under Appendix II of CITES was tabled at the CITES Conference of Parties (COP 11) in 2000. This proposal suggested that the trade of all *Harpagophytum* species needs to be regulated through an international instrument in order to curb potential unsustainable use. The DCWG thus played a major role in coordinating range states (countries in which devil’s claw is located), including Botswana, South Africa to respond to the proposal to regulate devil’s claw trade under CITES regulations.

The attention for INP development in Namibia did not only focus on sustainable practices for devil’s claw use. During the period between 1982 and 1999 interests also emerged for developmental options for other indigenous products. For instance, since 1992 a private enterprise Oontanga Oils Producer, pioneered the commercialization of cosmetic oil from Kalahari melon (*C. lanatus*). Gradually the value of INP products traditionally used by local communities got recognized, including oil for cosmetic products (mainly from *Ximenia americana* and *S. birrea*) and essential oils (from *Commiphora wildii*), which are used as fragrance for ointments (Nott and Curtis 2006). Consequently, it was deemed relevant to establish a multi-stakeholder governance body to coordinate the implementation of different INP development activities.

Initially attention focused mainly on fruit trees and in 2000 an Indigenous Fruit Task Team (IFTT) was formed under the Ministry of Agriculture Water and Forestry (du Plessis 2001; Schreckenberg 2003). The focus of this task team was subsequently widened to include all useful indigenous plants and the IFTT was changed accordingly to IPTT. Apart from fruits, herbs, resin, and nuts, interest was also shown in the commercialization of indigenous green leafy vegetables as a source of household nutrition. The IPTT became the central node for INP governance. The mandate of the IPTT is to coordinate sustainable commercialization of INPs in Namibia. Under the aegis of the commercialization action plan, much attention was given to developing a variety of pilot projects on propagation and domestication, chemical and nutrient analysis, development of technology and processing methods and marketing initiatives.

In fulfilling its commercialization roles, the IPTT often handles cases through which access to genetic resources of indigenous plants is arranged either to universities and research institutes or private entities. The way in which the IPTT dealt with these cases, contributed immensely to the national debate on access and benefit sharing, which was fueled by the CBD. Namibia had ratified the CBD in 1997—an international agreement, which requires that each contracting party creates conditions that facilitate access to genetic resources and its associated traditional knowledge. Thus, in 2007, an IBPC was established in Namibia to regulate access to genetic resources in the country. In fulfilling this international obligation of the CBD, the IBPC was established under the Directorate of Environmental Affairs of the Ministry of Environment and Tourism to serve as the competent national authority to facilitate access and benefit arising from the use of genetic resources. The CBD acknowledges that genetic resources and the associated indigenous knowledge can generate products of commercial value. When such products are accessed through mutually agreed terms and prior informed consent, the local and indigenous communities can benefit from the use of their knowledge, practices and innovations (Chennells 2009; Dutfield 2006). Within the context of the CBD, specific initiatives for access and benefit sharing were developed in Namibia for commercially promising INPs such as myrrh oil (*Commiphora* spp.), hoodia (*Hoodia gordonii*) (Wynberg 2004) and marula (*S. birrea*) (Ministry of Environment and Tourism 2010).

### Substantive Focus of the Different INP Governance Bodies

Due to the differences in their contextual background, the substantive focus of the three governance bodies varies. Whereas the DCWG focuses on developing the production, use and trade of the two *Harpagophytum* spp. i.e. *H. procumbens and H. zeyheri*, the IPTT focuses on many species from different genus. The IBPC does not focus on specific species, but on a specific policy issue, namely access and benefit sharing from the use of genetic resources. Also the mandates of the three governance bodies varies (Table 2). Whereas the IPTT was established to coordinate and facilitate exchange of knowledge, the DCWG and IBPC mainly focus on agenda setting and decision-making on specific policy issues.

**Table 2:** Mandates and representation in the INP governance bodies in Namibia

Governance bodies	IPTT	DCWG	IBPC
Policy mandate	Sustainable commercialization of all INPs in Namibia	Development of harmonized policies regarding the production and trade of devil’s claw	Provision of access to genetic resources as well as regulation and facilitation of bioprospecting activities in Namibia
	Exchange of resources and knowledge	Establish mechanisms for sustainable utilization and trade monitoring	
Coordinating Ministry	Ministry of Agriculture Water and Forestry.- Directorate of Research and Training	Ministry of Environment and Tourism-Directorate of Natural Resource Management	Ministry of Environment and Tourism-Directorate of Environmental Affairs
Actor representation			
Public sector	MAWF, MET, MTI, MoE, NAB	MAWF, MET and MTI	MAWF-NBRI, MET, MTI MoE-DRST, UNAM, MFMR-DRM, MSS, and the OAJ-DLA
Civil society organizations	CRIAA-SADC, NACSO, NNFU, NAU, CBOs, e.g. Ben Hur, Komeho Namibia, Eenhana TTC associations etc.	CRIAA-SADC, and IRDNC	Invited when necessary
Private sector	TTC, EWC, OOP etc. They participate as guest or observers with no voting rights.	Primary producers and private sectors not represented	Invited when necessary

*CBOs* community-based organizations, *CRIAA-SADC* Centre for Research Information Action in Africa-Southern African Development and Consulting, *DCWG* Devil’s Claw Working Group, *EWC* Eudafano Women Cooperative, *IBPC* Interim Bioprospecting Committee, *IPTT* Indigenous Plant Task Team, *IRDNC* Integrated Rural Development and Nature Conservation, *MAWF* Ministry of Agriculture, water and Forestry, *MAWF-NBRI* Ministry of Agriculture, Water and Forestry-Namibia Botanical Research Institute, *MET* Ministry of Environment and Tourism, *MET-DRM* Ministry of Environment and Tourism-Directorate of Resource Management, *MFMR-DRM* Ministry of Fishery and Marine Resources-Directorate of Resource Management, *MJ-DLA* Ministry of Justice-Directorate of Legal Advice, *MoE* Ministry of Education, *MSS* Ministry of Safety and Security, *MTI* Ministry of Trade and Industry, *NAB* Namibia Agronomic Board, *NACSO* Namibian Association of Community-based Natural Resource Management, *NAU* Namibia Agricultural Union, *NNFU* Namibia National Farmers Union, *OAJ-DLA* Office of the Attorney General-Directorate of Legal Advice, *OOP* Oontanga Oils Producers, *TTC* Tulongeni Twahangana Coorperative, *UNAM* University of Namibia

Among the three governance bodies, the IPTT plays the major role in INP development. The IPTT is mainly a multi-stakeholder platform for sharing resources and knowledge. Interviews showed that policymaking has not been one of the key roles for the IPTT. As a result of its robust membership, the IPTT has served as a catalyst for linkages between government and industry or government and interest groups, such as rural producers, research entities, and civil organizations. The IPTT members coordinate the implementation of the national INP action strategy. Based on this strategy, priority species for development are identified. Depending on the stage of product development and market trends, the IPTT identifies support measures for these priority species through an “adaptive pipeline approach”. In this approach, each product is given a different support. For instance, the products with emerging commercial potential are prioritized for research and development funding, while for those that already are being commercialized in self-sustaining value chains, such as marula oil, priority is given to developing stable commercial partnerships to maintain secure access to markets. Through its adaptive pipeline approach, the IPTT has played a significant role in product development and market research for several INPs, notably marula oil, Kalahari melon seeds, and ximenia oil from the seed kernels of *X. americana*.

In contrast to the IPTT, which focuses on several INPs, the DCWG deals only with devil’s claw. The DCWG serves as a platform through which knowledge on sustainable utilization and trade for this main Namibian commercial INP is shared. The DCWG also played a significant role in reviewing the national policies on the utilization of devil’s claw in Namibia and in harmonizing devil’s claw policies within the Southern Africa region by establishing a similar working group at regional level. At regional level, the working group was instrumental in the exchange of information and joint learning between South Africa, Botswana, and Namibia. This international collaboration mainly involves government ministries and research institutes and it provides for a wider scope of problem solution and adaptation through multi-level learning.

The IBPC was established with the specific aim of regulating, and facilitating bio-prospecting activities within Namibia. The IBPC is an interim body which represents the National Competent Authority on access and benefit sharing as required under the Nagoya Protocol of the CBD. Consequently, the jurisdictional status of IBPC is not only at national level but also at global level. At local level, the IBPC has played a major role in consultative workshops that preceded the development of the Bill on Access to Genetic Resources and its Associated Traditional Knowledge.

Unlike the IBPC that was established as an international obligation, the IPTT and DCWG arose from national initiatives. However, their scope became broader, with the IPTT becoming a member of Phytotrade Africa, which is an International Trade Association linking its members to the global market for natural products and pursuing new product development. The DCWG, in turn, has been replicated at the regional level through the creation of a Regional DCWG in 2002.

### Stakeholder’s Representation in the Governance Bodies

The history of the development of INP governance bodies is reflected in the configuration of the overall INP governance network. As illustrated in Fig. 3, the IPTT is the most important governance cluster with the greatest number of relations to other organizations, while the DCWG and IBPC have relatively fewer members. In addition to these specific INP governance bodies, other institutional structures also play an important role in facilitating collective decision-making and coordination of pilot projects for the commercialization of INPs in Namibia. One of the main nodal structures is the Centre for Research Information Action in Africa-Southern African Development and Consulting (CRIAA-SADC)—an instrumental NGO in INP development, which uses donor funds to provide INP services. This NGO also carries out consultancy work for the IPTT on issues related to INPs. In providing support to local communities, CRIAA-SADC has also served as an interim benevolent intermediary trader—sourcing INPs from user group associations and re-selling these products for export at cost recovery price. These arrangements mainly formed part of the pilot projects for the promotion of indigenous fruits.

In addition to CRIAA-SADC, the Ministry of Agriculture, Water and Forestry (MAWF) and the Ministry of Environment of Tourism (MET) serve as secretariat or chair to the three key governance bodies. The Directorate of Training and Research under the MAWF acts as the secretariat and chair to the IPTT, while the Directorates of Natural Resource Management and the Directorate of Environmental Affairs under MET serve as the secretariat for the DCWG and IBPC respectively.

The membership list demonstrates that the different governance bodies for INPs in Namibia are in principle relatively open to different interest groups of society. However, in practice the membership is limited either due to a lack of resources or due to the absence of a formal representative body. The public sector and civil society organizations are usually well represented, but the representation in the IPTT and DCWG forums of the private sector, community-based organizations (CBOs) and standardization bodies is minimal. Both the interviews and official proceedings indicate that the membership to both the IPTT and DCWG is mainly dominated by actors from the public sector and civil society.

Nonetheless, several efforts have been made to include CBOs in these governance bodies, in order to obtain representation reflecting different commercialized species and different ecological regions. Such efforts were facing several difficulties. For instance, the IPTT started an initiative to organize local INP harvesters and primary processing organizations (PPOs) into “eco-satellite regional centers”, which could act as nodes for rural development. These centers were incorporated into the IPTT as observers to enrich the IPTT forum with ideas and experiences from the different ecological regions. However, over time, only a few of these regional centers actively participated in the IPTT forums. The continuity in communication and exchange of information with eco-satellite centers was difficult to maintain due to regular staff turnover and changes in leadership at these centers. Another challenge concerned the financial means to enable staff of these satellite centers to travel from distant regions to attend IPTT forums.

Similar challenges of limited stakeholder participation in the governance process were experienced in the DCWG. Despite the original commitments to include multiple stakeholders (du Plessis 1999), the interviews and official proceedings indicate limited representation of primary producers, traders and exporters in the DCWG meetings. For traders and exporters, the absence of a formal trade association for devil’s claw has been a major stumbling block to gain trader representation in the DCWG. Recently (2014) a new initiative was undertaken to establish a Devil’s Claw Trade Association and this change is expected to provide an opportunity for traders and exporters to be represented in the DCWG. Traders could bring to the DCWG forum experiences of devil’s claw trade, including issues of product quality and price, as well as trade quota, thereby providing feedback to devil’s claw policy formulation process.

The experience of the IBPC governance body regarding participation of local stakeholders is different. Unlike other governance bodies, the IBPC has systematically invited traditional leaders, regional councils and CBOs to the consultative workshops they held prior to decision-making. Between 2011 and 2012 extensive regional consultative workshops were conducted in order to incorporate the views and ideas of local level stakeholders into the development of the Bill on Access to Genetic Resources and its Associated Traditional Knowledge. However, the opinions of respondents on these consultative workshops vary. Some key informant remarked that these workshops have created wrong and unintended expectations among some traditional communities. Some traditional authorities have started to demand access fees from bio-prospecting researchers before they grant access to their communities for bioprospecting.

This illustrates the difficulties involved in establishing local agreements on bio-prospecting and benefit sharing and the intricacies of developing proper arrangements for product commercialization. On the one hand, access to resources for research and innovation needs to be encouraged, but on the other hand safeguards are also needed to prevent biopiracy and maximize benefit sharing with local communities. It is critical to know where to strike the balance, such that the Bill does not frustrate research and innovation or provide loopholes to exploit local communities.

### Membership Interaction between Governance Bodies

The presence of the three major governance bodies is clearly visible in the overall structure of the INP governance network in the form of three key nodal points (Fig. 3). However, as illustrated by the network configuration, as the INP sector in Namibia is relatively small, the membership of the IPTT, DCWG, and IBPC is often overlapping.

This overlap in membership results in different levels of reciprocal exchange of knowledge and feedback. The IPTT meetings explicitly serve as a formalized platform for reciprocal feedback and sharing of information. This role is reflected in the regular sharing of the progress of DCWG activities with IPTT members. Such exchange of information is less frequent in the case of the IBPC, resulting into a state which is described by some respondents as inactive or dysfunctional. When interviewed, most IBPC members indicated a need to have more regular meetings in order to stimulate the sharing of practical experiences among the IPTT and IBPC members. A formalized reciprocal feedback between the IPTT and IBPC would also be instrumental for re-aligning the institutional framework for access to genetic resources and intellectual property rights issues, such as material transfer agreements, traditional knowledge protection, and farmers rights. Several interviewees also expressed the view that the IPTT contributed significantly to the visibility of Namibia at global level, which has led to Namibia being nominated as the key negotiator of the Nagoya Protocol in Africa. Due to this visibility, international development agencies, such as the Germany Society for International Cooperation (GIZ) and the Millennium Challenge Account (MCA) found the IPTT to be the best vehicle through which funding for INP activities can best be channeled.

## Functional Relations in the INP Policy Network

### Three Functional Clusters of Policy Development and Implementation

The INP policy network does not only illustrate the structural relations in terms of membership to the various specialized governance bodies, but also the functional relations between these actors. In Fig. 3 the functional relations can be distinguished as dotted circles around organizations that coordinate the different governance bodies (network managers) including the MAWF-NBRI, MAWF-DART, and MET-DRM. These clusters show the most frequently interacting organizations, which focus on specific functions, such as value addition, capacity building and development, and resource management.

The three sub-groups of actors focus on the following key functions:

1. Product quality involving product research and development, standardization and value addition;
2. Resource management and monitoring, including screening of useful botanical plants, propagation, cultivation, and domestication of indigenous species;
3. Institutional capacity building and development, which mainly involves training on harvesting techniques and good manufacturing practices for semi-processing procedures provided to harvesters and CBOs.

Cluster A consists of actors, such as the EWC, PTA including CRIAA-SADC. These actors regularly interact with MAWF-DART (see Fig. 3), and they are mostly engaged with activities in the field of value addition, product quality and standardization as well as product research and development. Within this cluster CRIAA-SADC has coordinated several pilot projects, often with financial support of international donor organizations. For instance, in order to stimulate the manufacturing of different marula products, attention focused on developing the extraction technology for pulp, juice, and flavor. Also, laboratory tests were conducted with funds from GIZ (German Development Cooperation) to analyze fatty acid profiles, microbial contaminants, and acid values of marula oils in order to develop food oils as a new product. Consumer trials were also conducted for different products. For instance, in an effort to maximize benefits to local communities, a community market arrangement was established between the Eudafano Women Cooperative (EWC) and the Body Shop International (BSI). A preferential access to market was granted to the EWC to supply BSI with marula oil. Another example in the area of value addition includes a pilot program on organic certification for devil’s claw under the SHDC project. This organic certification scheme was later replicated in communal conservancies in the Otjozonjupa Region. As a result of pilot certification, the proportion of organic devil’s claw products that was exported from Namibia during the period of 2003–2006 was estimated to range between 0.5 and 1.5% (Cole and Bennet 2007).

Cluster B consists of a group of organizations centered around MAWF-NBRI that are mainly involved in resource management, screening and monitoring. This cluster focuses on the creation of innovative systems for INP production. In developing new production systems, specific attention was given to INP propagation and cultivation, including domestication of indigenous species. The activities include surveys and screening of useful botanical plants; breeding and cultivation trials; as well as seed collection and nursery establishment. In implementing these activities, the MAWF-NBRI collaborated with various academic institutions and interest groups such as the Namibia National Farmers Union (NNFU) and the Hoodia Grower’s Association of Namibia (HOOGRAN). The DCWG commissioned the devil’s claw cultivation project to CRIAA-SADC with co-funding from the MAWF and European Development Funds under the Namibia Agricultural Sector Support Program (NASSP). In addition, the National Botanical Research Institute conducted breeding trials for Kalahari melons to improve seed oil quantity and quality, with co-funding from the MAWF and GIZ. The Kalahari melon trials were conducted in collaboration with the different agricultural research stations across the country in order to compare the influence of ecological variations on the performance of the melon.

The function of Cluster C, which is centered around the three key support organizations CRIAA-SADC, Namibia Nature Foundation (NNF) and Integrated Rural Development and Nature Conservation (IRDNC) has mainly focused on capacity building and development. The support organizations specifically work at local levels where they organize harvesters in PPOs and train them in various relevant skills such as leadership and organization management and basic business and marketing skills. The function of capacity building and development has largely been implemented by NGOs given the lack of capacity within government agencies. For instance, a project coordinated by the indigenous natural product component of the Millennium Challenge Account (MCA-N-INP) facilitated capacity building among INP harvesters. It is estimated that through the MCA-N-INP, about 9000 registered INP harvesters have been trained in various skills including sustainable harvesting of devil’s claw (MCA-N 2011a; MCA-N 2011b).

The network structure illustrates that pilot projects of INP commercialization have mainly been carried out by multiple organizations, each focusing on a specific function as illustrated in Clusters A, B and C. The focus of the functions of each cluster is often related to specific policy objectives. For instance, in response to the MET policy on biodiversity conservation, enrichment planting of indigenous plants in the wild is encouraged as a means to reduce harvesting pressure in wild areas that are closer to human resettlement. Similarly, the integration of INPs into agricultural farming systems is promoted as a way to implement the agricultural policy on poverty alleviation and income generation.

## Balancing the Power between State and Interest Groups

The discussions in the sections above demonstrate that the policy network for INPs in Namibia consists of different dimensions including governance bodies, actor representation, and functions of different governance clusters. Focusing on the IPTT as the main governance structure/body, its membership ranges from state, NGOs, societal interest groups (cooperatives, farmers union, producer associations etc.) and private entities. Such membership theoretically allows for balancing of power between the state and other stakeholders in decision-making. However, the Namibian experience illustrates that despite their common objective of promoting INPs, IPTT members have diverging interests and expectations, which lead to a certain degree of power imbalance in terms of deciding which course of action to undertake for the promotion of INPs. With support from NGOs, institutional arrangements have been established through which access to indigenous products is organized. These arrangements include trade cooperatives and exclusive purchase agreements that facilitate sustainable sourcing of INPs. However, the IPTT undertakes limited efforts regarding support and incubation of INP-based enterprises. For instance, the interviews with private small and medium enterprises (SMEs) in the cosmetic sector revealed that the IPTT does not meet their expectations. As upcoming business entities, SMEs expect the IPTT to disseminate information on standard specifications for different products, processing techniques and product formulations in order to stimulate value addition to natural products.

More specifically, Namibia has no entity with the capacity to filter some of the cosmetic oils that are exported by Namibian SMEs. The SMEs willing to add value (oil filtering and refining) have to export crude oil through South Africa, where the crude oil is filtered. In addition, there are other services that are beyond the capacity of an upcoming SME. For example, the SMEs require support in terms of training to primary producers in order to supply quality materials, marketing and promotion of natural products, and research and development. The SMEs expect that the IPTT provides some of these services. On the contrary, most of the information generated through the IPTT has been regarded as confidential information which can only be shared with an envisaged private holding company, structured to benefit primary producers. The idea of such a company did not materialize due to various criticisms. Some stakeholders, especially private entities, perceived that the holding company would compete with existing individual private entities and that it would be the sole beneficiary of research and development and market research that the IPTT has produced or commissioned with public funds. Other stakeholders, such as the public sector, did not support the establishment of a private holding company due to suspicions related to the proposed shareholding formula.

In the beginning, the IPTT largely focused on undertaking explorative studies in order to understand the context of the INP sector. These studies included the screening of botanical plants and identification of useful plants; breeding and cultivation of indigenous plants with known commercial values; development of extraction and processing technologies; consumer trials; and pilot organic certification. The IPTT commissioned most of these studies to NGOs and other professional experts at the higher education institutions. Some of the organizations that carried out these activities e.g. NGOs and the University of Namibia are also members of the IPTT. Thus the IPTT itself transformed into what has been referred to as an implementation and administrative body (Albertyn 2011), instead of a body that facilitates and coordinates activities. Also, the thrust on information sharing that was one of the key aims of IPTT establishment gradually diminished. Most respondents highlighted that the roles and functions of the IPTT were ambiguously understood by both the general public and some IPTT members. In the opinions of some IPTT members, explorative studies that pioneered the different functions of the INP value chains have dominated the functions of the IPTT at the expense of tangible poverty alleviation activities. The explorative studies have been described as being too ambitious, stretching the IPTT into too many functions with limited financial and human resources. The key stakeholder—the Ministry of Agriculture Water and Forestry (MAWF)—also perceived that the outcomes generated by the dominance of explorative studies generated few tangible benefits to local communities that could contribute to poverty alleviation such as technical capacity empowerment, value addition and fair product prices.

The audit commission that was established to re-strategize and re-focus the functions of the IPTT in 2011, revealed how the MAWF—which can be regarded as the network manager of the IPTT—proposed a new direction for the IPTT. The audit report indicates that enough data collection and explorative studies have been conducted and there is need to focus more on empowering local level primary producers and infrastructure development to enable value addition. This implies that the interests of professionals and experts in explorative studies dominated the focus of IPTT activities at the detriment of other objectives such as support to SMEs and trade cooperatives.

To some extent the DCWG and the IBPC, which are dominated by government actors, considered the needs and interests of different sectors of society. For example, the proceedings of the devil’s claw workshop that was held in 2002 demonstrate how the government (MET), despite its interest in biodiversity conservation, eventually did not support the proposal to list devil’s claw under Appendix II of CITES out of recognition of the interests of NGOs and harvesters in promoting devil’s claw trade to support rural livelihoods.

## Impact of the Governance Structure on Policy Learning Processes

The multidimensional policy network for INPs in Namibia has greatly facilitated a learning process in respect to both policy formulation and implementation. This involves interactions between different stakeholders engaged in both policy development and pilot project implementation.

### Learning Process for Policy Formulation

An example of changes in the policy is demonstrated through the DCWG, which facilitated a learning process and policy formulation by tapping from experiences on devil’s claw pilot projects. Initially, under the provisions of Schedule 9 of the Nature Conservation Ordinance (4 of 1975), a permit is required for harvesting (gathering), purchasing and trade of devil’s claw. The harvesting permit was issued to individuals for a duration of 1 month and there was no clear control of harvesting localities and quota (Motlhaping 1999). Due to a lack of capacity to monitor resources and inspect the harvesting activities, legal provisions of the harvesting permit was abandoned since 1986, leaving only the export permits in force (Hamunyela 1999). A re-assessment of the situation on devil’s claw harvesting took place in 1999, stimulated by national concerns about overharvesting and unsustainable harvesting methods. Consequently, a draft policy to control harvesting and trade of devil’s claw was developed, later culminating into a National Policy on Utilization of Devil’s Claw (*Harpagophytum* spp.) Products, which was approved in 2010.

The policy was adapted to enable the traceability of devil’s claw products, to monitor harvesting localities and quantities, and to extend the harvesting duration from 1 month to 8 months (March–October). The new policy also made it possible for user group associations to acquire a collective group permit which enhances collective management and self-regulation at local level. Group permits are issued by MET only when approval is granted by a local authority or by the land owner where devil’s claw will be harvested. Establishment of these exclusive access rights to harvesting areas discourages the earlier open access to lands and enhance the organization of harvesters in user group associations where they are trained in sustainable harvesting skills. Regulated access to devil’s claw harvesting sites thus stimulates sustainable harvesting practices.

Under the new policy, the registered harvesters need to report to MET the quantities harvested, stipulating locations where the resources were harvested. This information is passed to the buyer of devil’s claw, who is required to document the purchase transaction (source and quantity of all purchased devil’s claw). The development of new policy regulations was greatly stimulated by various efforts aimed at coordinating harvesting practices to develop sustainable harvesting techniques. The impact of these changes is demonstrated by an observed increase in the proportion of SHDC products from 48% in 2008 to 55% in 2011 (Moller 2013).

In addition to these policy changes at national level, the DCWG as a policy network has also influenced policy processes at regional and international level. Notably the experiences of the devil’s claw policy review has significantly shaped discussions at the CITES Conference of Parties during the period between 2000 and 2007. At this conference, the preferred strategy for devil’s claw policy in Southern Africa was articulated. Between 2000 and 2007 discussions took place within CITES structures whether to regulate devil’s claw harvesting under CITES Appendix II of endangered species—whose trade should be controlled in order to conserve the species. However, within Namibia the policy regarding devil’s claw was not only drawing from CITES regulations, but was also guided by the provisions of the Nagoya Protocol of the CBD on traditional knowledge and benefit sharing (CRIAA SADC Namibia 2002).

Following these dynamics at national level, the DCWG advocated a self-regulation approach instead of CITES regulations in the Southern African producer countries (South Africa, Botswana). The self-regulation approach is focused on creating incentives for sustainable utilization and improvement of people’s livelihoods. Specifically, this approach promotes development of benefit-sharing mechanisms to generate profit from indigenous knowledge (Dickson 2008). Eventually, the Namibian experiences with the new policy practices for stimulating sustainable utilization of devil’s claw were among the cases that were instrumental in influencing CITES’ approach to international trade regulation of endangered species (Dickson 2008).

Funding organizations have also influenced the changes in devil’s claw policy. Specifically, when the MCA-N project negotiated the terms of the project operation, it was initially agreed that INP development activities under the project will only commence once the proposed domestic legislation on Access to Genetic Resources and its Associated Traditional Knowledge is approved by the parliament. This legislation was however delayed because the Nagoya Protocol on Access and Benefit Sharing was not yet approved. Later, the conditions for the MCA-N project on INPs were narrowed to other policy measures including granting a protection status to all *Harpagophytum* species in Namibia.

### Learning Processes for Policy Implementation

Unlike the clearly visible changes in the content of the devil’s claw policy, the changes on other INPs in Namibia mainly concern changing institutional practices, but not policy content. In particular, important changes are gradually observed regarding value addition and product quality standardization as a strategy toward poverty alleviation. New practices emerged through the industrial policy interventions, which are coordinated under the Ministry of Trade, Industrialization and SME Development formerly known as the Ministry of Trade and Industry (MTI). Since 1997, the MTI has embarked on a program to support and strengthen the capacity of SMEs by providing processing equipment, financial assistance and subsidized industrial business outlets in order to equip them with the necessary capacity for value addition to natural products. The idea of value addition is also evidenced by the upgrading of agricultural laboratories at the MAWF, established to develop skills in analyzing biochemical and chemical properties of agricultural and natural products. The idea of value addition is also reflected in the gradual emergence of small-scale processing facilities. In 2004 a small-scale marula factory was established for the extraction of marula crude oil and marula juice in the Oshana Region close to the source of marula nuts. In 2010 a small-scale distillation factory for the extraction of essential oil from *Commiphora* resin was also established in the Kunene Region where *Commiphora* resin is harvested. Plans are also underway to support the communities in the Ohangwena Region with the establishment of a processing facility for ximenia oil.

## Discussion

The aim of this paper was to assess the nature and complexity of the INP governance network in Namibia by analyzing the structural patterns of relations between actors and to explain how these structures influenced INP policy formulation and implementation. The findings indicate that the network is characterized by the presence of three specially created INP governance bodies, each having a specific policy mandate. The network structure also shows the presence of other forms of governance clusters, which are also central to INP governance as they focus on distinctive functions.

The findings show several critical issues that need further attention. A major issue concerns the dominance of representatives from the formal state bureaucracies, NGOs and academic/research institutes and the limited representation of locally based primary producers. According to Jordan and Schubert (1992), the policy network may restrict membership or maintain a high threshold of access. In a restricted policy network, the agenda for policy development is dominated by the interest groups that are represented in the network (Schneider 1992). The IPTT as a policy network for INPs mirrors this form of a network with specific issues dominating agenda setting. As described in the “Results” section, the IPTT functions have been dominated by explorative studies and botanical plant screening. Whereas this is a relevant function for pioneering the commercialization of INPs, it has overwhelmed other functions, such as exchange of information and empowerment of SMEs for value addition.

Although the establishment of the multi-stakeholder forum IPTT has been admired as an exceptional strategy in Southern Africa (Laird et al. 2010), the lack of representation of local actors brings with it a danger of overlooking the relevance of local level issues and constrains to policy implementation by restricting wider collective actions (Klijn and Koppenjan 2000). To allow incorporation of issues on the ground, a well-balanced network which links local communities to the national decision makers is needed in order to minimize power imbalances, which is an inherent characteristic of policy networks (Marsh and Rhodes 1992). According to Laird et al. (2010, p. 347), participation of harvesters in the policy-making process is often limited by the lack of producer organizations, which serve as an institutional vehicle through which concerns of the harvesters can be channeled. In contrast, Namibia has embarked on the establishment of producer organizations, locally known as Primary Processing Organizations (PPOs). Limited representation in Namibia is associated with a lack of resources to maintain linkages between PPOs on the ground and the IPTT forum.

To prevent such power imbalances, Klijn and Koppenjan (2000) suggest that it is necessary that public actors provide leadership in optimizing the conditions for interaction among actors. The assumptions are that public actors pursue interests of all members of society. The public actor can engage in active network management giving continuous attention to network constitution (bringing in new actors) and changes in the rules of interaction (Klijn and Koppenjan 2000). The experiences with INP commercialization in Namibia support this crucial role of network management by public organizations, such as the MAWF, MET, or the then Ministry of Trade and Industry (MTI). The ban on the export of Kalahari melon seeds by MTI illustrates the relevance of such public leadership. Despite an established market for raw seeds, seed export was banned as an endeavor to promote value addition by small and medium manufacturing firms and to increase their competitiveness (Schreckenberg 2003).

However, our analysis also indicates that the actor constellations are shifting as project-related civil society organizations are taking on traditional government roles. This is specifically reflected in the dominant role of NGOs facilitating INP pilot projects in Namibia. Similar observations have been reported from the Congo Basin (Ingram 2017) where most efforts to promote the production of NTFP are spearheaded by development projects. This indicates that there is a need to give further attention to the provision of platforms that enable balancing stakeholder interests and power in such project-driven governance networks characterized by a variety of thematically oriented civil society organizations and private sector organizations.

Our analysis further reveals that power imbalances remain due to limited representation of harvesters in the IPTT and DCWG networks. This suggests that these networks could be further strengthened by improving their role as a platform for sharing knowledge and information between all relevant stakeholders (Albertyn 2011). Further attention still needs to be given to organizing discussion forums in which participants can share ideas and develop common views on the problem and associated solutions and goals (Schon and Rein 1994). More interactions between the different stakeholders at local, national and even international level and between the different governance bodies would stimulate further collaboration and interaction between a varieties of governance practices and contribute toward shared goals and mutual trust among all relevant groups of actors.

## Conclusion

The establishment of the IPTT as a central governance body for INPs in Namibia has provided a well-articulated multi-stakeholder platform for resource mobilization and knowledge exchange. In combination with the activities of the DCWG, IBPC and other forms of governance clusters identified, the structure of the INP policy network influenced the execution of a series of activities in the fields of policy formulation and implementation.

Although this multi-dimensional governance structure has had a noticeable influence on the INP policy formulation and implementation processes, there is still a need for further improvement. In the first place, further attention is needed to actor constitution and rules of interaction. In order to allow discussions that yield policy feedback to a full array of issues of interest to a range of stakeholders, further widening of the participation of harvesters, CBOs and private companies is required. If necessary, the IPTT may also facilitate the establishment of separate entities, such as the much-needed society of cosmetics industries that can provide necessary information to cosmetic SMEs. Specifically, the IPTT needs to pay attention to the functions of the value chain for different enterprises, such as SMEs producing cosmetics, fragrance or herbal remedies in order to address issues of interest to particular INP enterprises.

Increased attention also needs to be given to policy adjustments based on the experiences of various explorative studies and pilot projects that have been conducted so far. This requires not only increased research and evaluation of pilot projects, but also the mobilization of specific development issues that are relevant to specific ecological regions or for the promotion of INPs. For example, policymakers need to find new directions after reaching a deadlock on the proposed ownership model for INP enterprises in Namibia.

Finally, our analysis illustrates that the complexities of INP governance can be analyzed through a policy network lens. The policy network approach elucidated the diversity and complexity of INP actors, their different functions, and the needs of different enterprises and ecological regions.
